# The effect of N95 designs on respirator fit and its associations with gender and facial dimensions

**DOI:** 10.1371/journal.pone.0288105

**Published:** 2023-11-29

**Authors:** Nurul Amalina Khairul Hasni, Rohaida Ismail, Rosnawati Muhamad Robat, Nadia Mohamad, Fatin Amirah Suib, Muhammad Alfatih Pahrol, Haalah Mahmud, Baderin Osman, Yin Cheng Lim, Zamtira Seman, Rafiza Shaharudin

**Affiliations:** 1 Environmental Health Research Centre, Institute for Medical Research, National Institutes of Health, Ministry of Health, Malaysia; 2 Infectious Disease Research Centre, Institute for Medical Research, National Institutes of Health, Ministry of Health, Malaysia; 3 National Institute of Occupational Safety and Health, Ministry of Human Resources, Malaysia; 4 Department of Social and Preventive Medicine, Faculty of Medicine, University Malaya, Kuala Lumpur, Malaysia; 5 Sector for Biostatistics & Data Repository, National Institutes of Health, Ministry of Health, Malaysia; VIT University, INDIA

## Abstract

This study examined the association of various brands of NIOSH-certified N95 filtering face-piece respirators (FFR) fit with facial dimensions and gender. One hundred and thirty-five participants (77 females and 58 males) were recruited from the previous facial anthropometry study among Malaysians in 2020. Quantitative respirator fit testing of six FFR were performed using the TSI Portacount Pro+ 8038 which comprised of four exercises (bending over, talking, up-down head movement, and side to side head movement). An overall fit factor (FF) of ≥ 100 was considered a pass for each FFR. Analysis was done using T-test, Pearson’s correlations, and generalised linear regression. The passing rates for the six FFR were 36.3% (Cup B), 50.4% (Trifold A), 54.1% (Duckbill A), 57.0% (Cup A), 74.1% (Trifold B), and 83.7% (Duckbill B). Both Duckbill B and Trifold B had the highest passing rates for both genders. However, certain FFR models (Cup B, Trifold A, Trifold B, and Duckbill A) fit better for participants with large facial size who were mostly males, while others (Cup A and Duckbill B) specifically fit better for those with small facial size, who were mostly females. This study showed significant positive effect of nose protrusion, nasal root and subnasale-sellion and the negative effect of menton-sellion, bigonial breadth and nose breadth on fit factors of various FFR. The results of this study emphasized the importance of choosing and designing FFR based on local anthropometry data, with careful consideration on the dimensions that affect the respirator fit. Since N95 are commonly used in the healthcare settings to prevent airborne transmission, the practice of respirator fit testing and selecting N95 with high passing rates for healthcare workers need to be emphasized.

## Introduction

The recent coronavirus disease 2019 (COVID-19) pandemic created a major demand for usage of personal protective equipment (PPE), especially respiratory protection device, in healthcare and community settings. Filtering facepiece respirators (FFR) are specifically designed to ensure respiratory protection against airborne contaminants including droplets and airborne infections. As a tight-fitting respirator, negative pressure is generated by the user inside the facepiece during inhalation, compared to the ambient air pressure in the environment [1]. N95 is a type of FFR that is widely used in healthcare facilities to protect healthcare workers from infectious respiratory diseases such as tuberculosis, severe acute respiratory syndrome (SARS), influenza, Middle East respiratory syndrome (MERS), mumps, and other droplets- or airborne-transmitted diseases [2]. The emergence of COVID -19 has only underscored the importance of N95 usage. The Centres for Disease Control and Prevention (CDC) of the United States (US) recommends the use of N95 when treating COVID-19 patients, while the World Health Organization (WHO) recommends the use of N95 when performing aerosol-generating procedures on COVID-19 patients [3, 4]. However, cross-contamination and nosocomial infection have been reported although healthcare workers wore full personal protective equipment when handling infected patients [5]. One of the causes could be attributed to the effectiveness of the FFR in filtering the airborne particles. This will depend on how well it fits the user’s face, which in turn determines whether the FFR seals sufficiently [6, 7]. A FFR with poor fit would reduce its protective performance and increase the risk of respiratory transmission.

As there is a wide range of facial sizes and shapes in various combinations among individuals, it is unlikely a single FFR will fit all [8]. The design and size of FFR should always be considered, as it may fit some users perfectly while leak for others [9–11]. The CDC reported that a mismatch between the FFR and the facial structure of healthcare workers resulted in a 6–88% failure rate in respirator fit and a 33% reduction in protective efficacy against infectious agents [12]. The type, model, and size of FFR fit differently according to facial dimensions, which can be influenced by gender, ethnicity, and age groups [13–15]. Facial dimensions, such as facial length and width, are some of the significant variables in a FFR’s proper fit [16]. According to Oestenstad et al. (2007), the bigonial breadth and menton-nasion length were the most important dimensions related to respirator fit among American participants [17]. On the other hand, Han and Choi (2003), concluded that the face width, bitragion-menton arc, and nose protrusion should be measured as a critical dimension in the development of respirators for Koreans [18]. Other studies done in the US and United Kingdom (UK) showed that Asians or members of minority groups were noted to have a lower success rate in fit testing compared with Caucasians [11, 19, 20]. These findings highlighted how the differences in facial dimensions among different ethnicities had affected respirator fit. Therefore, facial dimensions should be considered as a main factor in choosing N95 for a multi-ethnicity population such as Malaysia. With regard to gender, a few studies found that males were consistently associated with higher fit test passing rates for FFR [18, 21–23]. A meta-analysis by Chopra et al. (2021) showed that facial anthropometrics were varied between gender and ethnicity, which resulted to lower fit test passing rates among females and non-white cohort, particularly Asians [24]. In contrast, findings from a US study among white American participants found that there were no gender differences in respirator fit [17].

Ideally, users of a particular FFR should be fit tested annually by qualified personnels. The fit test should also be performed after significant facial changes such as weight gain or weight loss, dental procedures, scarring, or facial surgery [25, 26]. The test can be performed using either qualitative or quantitative methods. According to the Occupational Safety and Health Administration (OSHA) of the United States Department of Labor, qualitative fit test is a pass or fail test that assesses the adequacy of respirator fit through individual’s response to the test agent, whereas quantitative fit test assesses the adequacy of respirator fit by numerically measuring the amount of leakage into the respirator [27]. A review by Regli et al. (2020) recommended the use of the quantitative method as it has been shown to detect leakage better in previous studies [28]. Another important part of a fit test is seal check, which is required to be performed by the users before using a FFR [25]. One study found that seal check should be performed daily to ensure no leakage and proper fit especially when performing aerosol-generating procedures [5]. Regular practice of seal check has shown to be effective in increasing the fit test passing rate [29]. Besides, factors such as the presence of facial hair, adjustability of the straps, and positioning of the FFR may also influence the proper sealing of FFR and their passing rates [28].

Malaysia does not produce our own FFR. Most FFR were imported from the US and China. Based on available studies, the US and China’s population have different facial dimensions and facial panels compared to Malaysians [30, 31]. With the assumption that the produced FFR were based on their facial panels, the imported FFR will most likely unable to fit well with our population. Studies conducted in Asian countries such as Iran and China also showed low passing rates (2.7%–63.5%) when using the imported respirators [10, 32]. Besides, other studies have shown that fit test passing rates were especially lower among women and Asians which could be due to incompatibility of the FFR in relation to facial dimensions [16, 19, 22].

Although Malaysia refers to the Occupational Safety and Health (Use and Standards of Exposure of Chemicals Hazardous to Health) Regulations 2000 and the policies on infection control in healthcare settings has outlined the need for fit testing procedure [33, 34], the implementation of fit testing is lacking in most centres. Most healthcare facilities in Malaysia supply universal size FFR to their healthcare workers. However, with the lack of fit testing procedure to assess the efficacy of FFR, they are at high risk of exposure to airborne biological hazards such as COVID-19. In view of this, it is important that our healthcare workers were given the appropriate FFR that fit well to be adequately protected from infectious agents. They should also receive regular training and monitoring to ensure proper donning of respirators [35, 36]. Therefore, this study aimed to fill this gap by determining the best respirator fit of various N95 with different designs according to facial dimensions and gender. In particular, the role of quantitative fit testing in healthcare settings in choosing the best N95 for workers can be emphasised. Importantly, the result from this study, which is based on Malaysian population with diverse range of facial dimensions, can be applied for Malaysian healthcare workers who work in high-risk settings.

## Methods

### Participants

**Fig 1:**
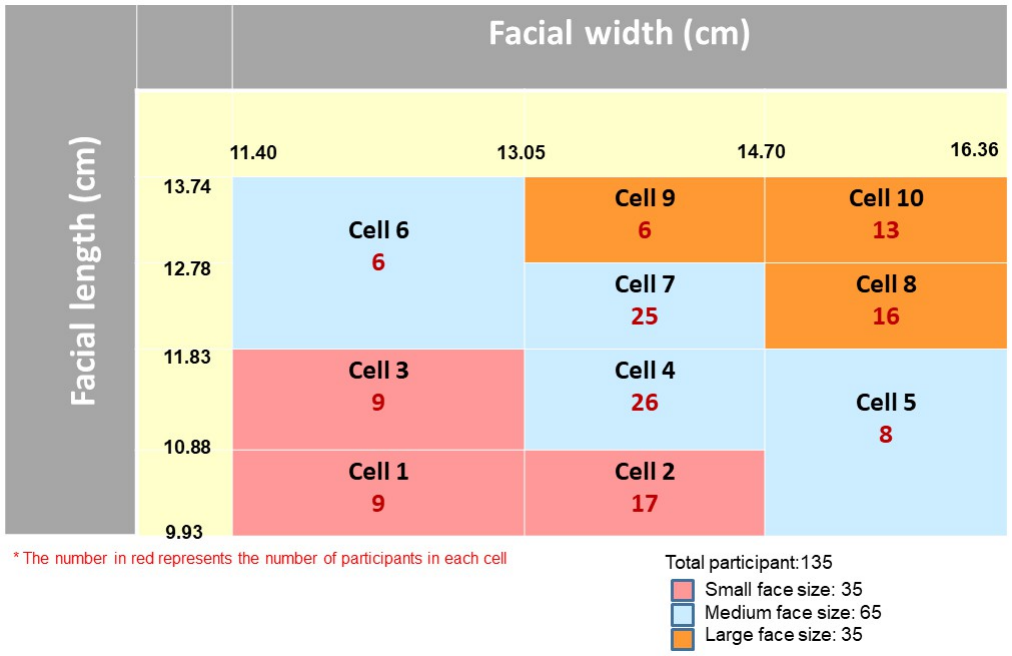
Distribution of participants based on the Malaysian bivariate facial panel.

Between December 2021 to January 2022, a total of 135 participants from the states of Selangor, Kuala Lumpur, Putrajaya, Melaka, and Negeri Sembilan were recruited to participate in this study. They were randomly selected from a subset of respondents from a previous study conducted in 2020 that collected facial anthropometry data among Malaysians [30]. Participants were selected based on their facial cells (cell 1–10) and sizes (small, medium, and large) based on the Malaysian bivariate facial panel derived from Malaysia’s facial anthropometry data [30], which was categorised by their facial length (menton-sellion length) and facial width (bizygomatic breadth) (Fig 1). Facial cells 1, 2, and 3 were categorised as small; 4, 5, 6, and 7 as medium; and 8, 9, and 10 as large facial size. The distribution of small, medium, and large facial size samples was 35, 65, and 35 participants respectively.

Inclusion criteria for the selection of participants were, age at least 18 years, good physical health, and ability to follow instructions. Male participants were required to shave off beard, and participants with symptoms of respiratory infection were excluded from this study.

This study was approved by the Medical Research and Ethics Committee (MREC), Ministry of Health Malaysia [KKM/NIHSEC/P21-1160(9)]. Written informed consent form were signed by subjects who agreed to participate. COVID-19 Rapid Test Kit Antigen (RTK-Ag) testing was also performed by all investigators and participants before each fit testing session to prevent the transmission of COVID-19.

### Respirators

A total of six NIOSH—certified N95 available in the Malaysian market and commonly used in the medical industry, especially by healthcare workers were selected for this study. Different designs of FFR were selected with two of each representing the cup, trifold, and duckbill shaped. All subjects were asked to perform a fit test for each of the selected FFR.

### Fit testing protocol

Quantitative fit testing was performed using the TSI Portacount Pro+ 8038 respirator fit testers. The experiment was conducted in accordance with the modified Condensation Nuclei Counter (CNC) protocol in OSHA 29CFR 1910.134 [37]. The protocol included four exercises (bending, talking, turning the head side-to-side, and moving the head up-and-down) for a duration of 2 minutes and 29 seconds including ambient sample collection. In order to obtain reliable results, participants had to be physically healthy and were not allowed to smoke cigarettes or cigars, drink or eat anything for at least 30 minutes before the test started.

Fit testing was conducted in a room with either operating air conditioning systems or natural ventilation, depending on availability. A sodium chloride (NaCl) particle generator (model 8026, TSI Inc., USA) was used to increase the ambient air concentration levels, mostly in the morning or at the beginning of the fit test. The particle generator was placed at least six feet away from the respirator fit tester during operation. Ambient concentration was measured by conducting “Daily Checks” each time before performing fit testing.

Participants were instructed on how to properly don and doff the FFR. The FFR were donned five minutes before starting the test to ensure participants were comfortable, and to allow them to adjust the respirators and remove any trapped particulates. Then, participants were instructed to perform seal check each time they put on an FFR. During the exercises, participants were not allowed to adjust the FFR to avoid invalid results.

The overall fit factor was obtained from the calculations of harmonic mean of every exercise’s FF recorded at each test. The tests were considered passed if they achieved the minimum required FF for FFR, which is ≥100.

Calculation of fit factor [38]:

$$FF=\frac{C_{B}+\;C_{A}}{2C_{R}}$$

C_B_ = particle concentration in the ambient sample before the respirator sample

C_A_ = particle concentration in the ambient sample after the respirator sample

C_R_ = particle concentration in the respirator sample

Calculation of overall fit factor:

$$Overall\;FF=\frac{n}{\frac{1}{{FF}_{1}}+\;\frac{1}{{FF}_{2}}+\;\frac{1}{{FF}_{3}}+\;\frac{1}{{FF}_{4}}}$$

where: *FF*_x_ = fit factor for test cycle

*n* = number of test cycles (exercises)

### Statistical analysis

Data were analysed using the Statistical Package for the Social Sciences (SPSS) version 28 (SPSS Inc. Chicago). Patient’s demographic characteristics and head-and-face anthropometric dimension, retrieved from previous study [30] were analysed as simple descriptive of frequency (percentage) and mean (standard deviation), stratified by gender. Crosstabulation and clustered column were then applied to assess their frequency distribution with passing rate and FF. Geometric mean and geometric standard deviation of overall FFs were also calculated by respirator type for both males and females. FFs were log-transformed and then averaged across subjects due to small positive skew and were usually log-normally or near log-normally distributed [39]. This new average was then inverted back to real value. Independent t-test was performed to determine the mean difference between fail and pass result of each facial dimension by types of FFR. Pearson’s correlation and generalized linear model of gamma with log link was then applied to analyse the correlation and magnitude of effects between facial dimension towards the fit factors of respirator type. The results with p-value <0.05 will be considered as having significant association.

## Results

### Demographic and head-and-face anthropometric dimensions

All 135 recruited participants had completed the quantitative respirator fit test for six FFR individually, with a total of 810 fit tests performed. Of the 135 participants, 58 were male and 77 were female. The age range of participants was 18 to 70 years old with a mean (SD) age of 38.36 (14.69) years. The body mass index (BMI) range was between 16.0 and 43.4. Most subjects were classified as overweight (58.5%), followed by normal weight (34.8%) and underweight (6.7%) (Table 1).

**Table 1:** Distribution of samples by demographic and facial dimensions.

Variables	Females = 77 n (%)	Males = 58 n (%)	Total = 135 n (%)
**A. Demographic**			
Age (years) Mean (SD)	38.55 (15.12)	38.10 (14.22)	38.36 (14.69)
25 and below	20 (26.0)	12 (20.7)	32 (23.7)
26–40	27 (35.1)	21 (36.2)	48 (35.6)
Above 40	30 (39.0)	25 (43.1)	55 (40.7)
BMI (kg/m^2^) Mean (SD)	26.70 (6.59)	28.41 (6.28)	27.44 (6.49)
Underweight	7 (9.1)	2 (3.4)	9 (6.7)
Normal	29 (37.7)	18 (31.0)	47 (34.8)
Overweight	41 (53.2)	38 (65.5)	79 (58.5)
Facial Size			
Small	32 (41.6)	3 (5.2)	35 (25.9)
Medium	39 (50.6)	26 (44.8)	65 (48.1)
Large	6 (7.8)	29 (50.0)	35 (25.9)
**B. Facial Dimensions** (cm) Mean (SD)			
Bigonial breadth	10.80 (1.12)	12.12 (0.96)	11.37 (1.24)
Bizygomatic breadth	13.66 (0.93)	14.63 (0.99)	14.08 (1.07)
Menton-Sellion length	11.26 (0.73)	12.54 (1.01)	11.81 (1.07)
Head breadth	15.32 (0.67)	16.00 (0.71)	15.61 (0.76)
Interpupillary breadth	6.24 (0.38)	6.83 (0.64)	6.49 (0.59)
Frontal breadth	9.46 (0.91)	10.15 (1.36)	9.76 (1.18)
Nasal root	1.73 (0.20)	1.84 (0.24)	1.78 (0.22)
Nose breadth	3.99 (0.31)	4.48 (0.57)	4.20 (0.50)
Nose protrusion	1.66 (0.21)	1.78 (0.21)	1.71 (0.22)
Subnasale-Sellion length	4.71 (0.46)	5.07 (0.41)	4.86 (0.47)

It is also apparent that participants with small facial sizes were predominantly females while those with large facial sizes were mostly males. The mean measurement for all facial dimensions was higher in males compared to females across all dimensions.

### Fit factor and passing rate

The passing rates of the six FFR for all subjects were summarized in total and according to gender (Table 2). There were two models for each type of FFR’s design (cup, trifold and duckbill). Overall, the passing rates ranged between 36.3% and 83.7% with an average passing rate of 59.0% for all six FFR. From 135 participants, 10 participants passed the fit test for all six FFR meanwhile, six participants failed for all. Duckbill B had the highest passing rate followed by Trifold B, Cup A, Duckbill A, Trifold A and Cup B. According to gender, Duckbill B, Trifold B and Trifold A were the most effective FFR among male participants. Meanwhile, Duckbill B, Trifold B and Cup A were the most effective for female participants. This is also similarly reflected in the geometric mean of overall fit factors for each FFR brand (Table 3).

**Table 2:** Passing rate for N95 according to gender.

Respirator	Passing rate		
	Male (N = 58)	Female (N = 77)	Total (N = 135)
	n (%)	n (%)	n (%)
Cup A	56.8	57.1	57.0
Cup B	37.9	35.0	36.3
Trifold A	62.0	41.5	50.4
Trifold B	79.3	70.1	74.1
Duckbill A	56.8	51.9	54.1
Duckbill B	82.7	84.4	83.7

**Table 3:** Geometric mean (GM) of overall fit factors (FF_Ο_) of N95.

Respirator	GM FF_Ο_ (GSD)			Min FF_Ο_	Max FF_Ο_
	Male	Female	Total		
Cup A	84.14 (2.41)	76.31 (2.83)	79.64 (2.65)	5	200
Cup B	47.00 (3.31)	31.16 (4.95)	37.29 (4.27)	1	200
Trifold A	92.61 (2.78)	70.77 (2.70)	79.60 (2.75)	3	200
Trifold B	131.44 (2.20)	112.08 (2.75)	122.24 (2.50)	3	200
Duckbill A	80.18 (2.99)	69.11 (3.44)	73.75 (3.25)	3	200
Duckbill B	140.08 (1.87)	148.54 (2.02)	144.79 (1.95)	4	200

**Fig 2:**
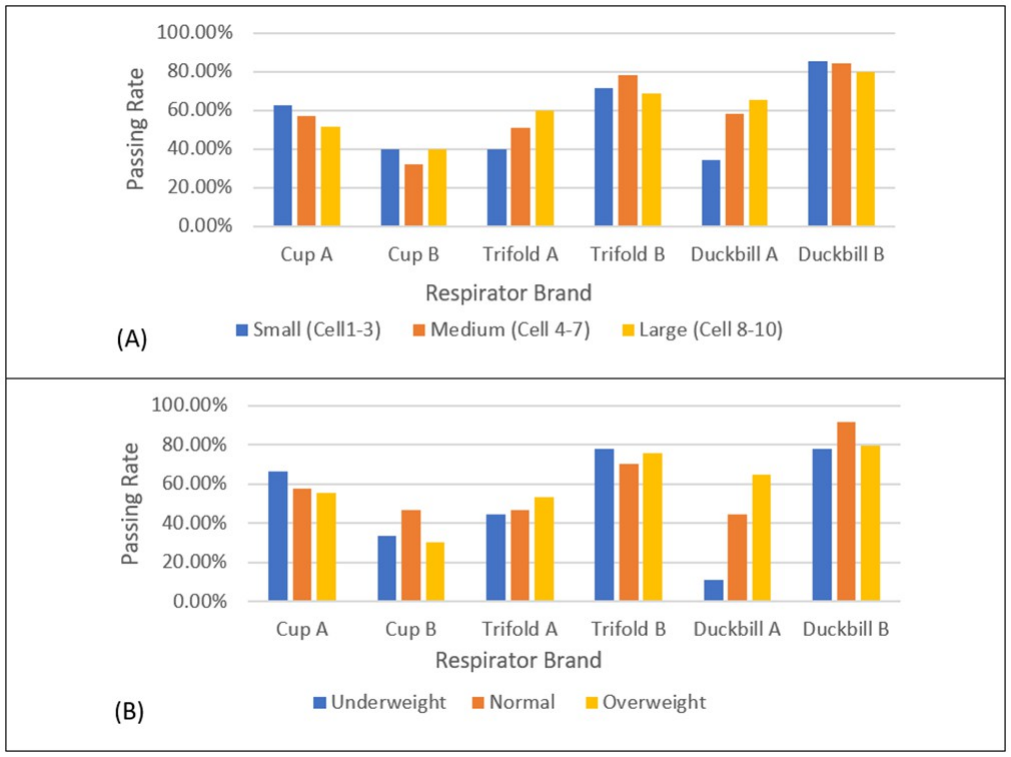
(A) Fit test passing rate based on facial size. (B) Fit test passing rate based on BMI.

Amongst all FFR, Duckbill B and Trifold B had the highest passing rates across all facial sizes and BMI classifications (Fig 2). Cup designs in this study are the only FFR that comes in two sizes, small (Cup A) and regular (Cup B). Although it was meant to cater for the different facial sizes, the smaller Cup A performed better across all comparison groups compared to Cup B. It can also be noted that FFR with similar designs produced different passing rates across all comparison groups with one model being much superior than the other (Table 2 and Fig 2).

The comparisons of facial dimension measurements were based on the test results of either ‘pass’ or ‘fail’ (Table 4 and S1 Table). Independent T-test performed on means of fit factor for all subjects showed significant differences (p<0.05) between ‘pass’ and ‘fail’ for Cup A for the following dimensions; menton-sellion length (t = -0.736; p = 0.027), interpupillary breadth (t = -0.844; p = 0.037), and nose breadth (t = -0.893; p<0.001). Meanwhile, for Cup B and Trifold A, significant differences were seen for subnasale-sellion (t = 0.202; p = 0.007) and frontal breadth (t = 0.847; p = 0.024) respectively. Other dimensions for the rest of the FFR were not significantly different (p>0.05). Among these three FFR, those who passed the fit test for Cup A had smaller measurement for almost all facial dimensions compared to those who passed for Cup B and Trifold A.

**Table 4:** Facial dimensions of participants according to ‘pass’ and ‘fail’ test results for each N95.

**Total (N = 135) Dimension (cm) mean±SD**	**Cup A**		**Cup B**		**Trifold A**		**Trifold B**		**Duckbill A**		**Duckbill B**	
	**Pass (n = 77)**	**Fail (n = 58)**	**Pass (n = 49)**	**Fail (n = 86)**	**Pass (n = 68)**	**Fail (n = 67)**	**Pass (n = 100)**	**Fail (n = 35)**	**Pass (n = 73)**	**Fail (n = 62)**	**Pass (n = 113)**	**Fail (n = 22)**
Bizygomatic breadth	14.03 ± 1.00	14.15 ± 1.15	14.09 ± 1.06	14.07 ± 1.07	14.15 ± 1.07	14.01 ± 1.06	14.11 ± 1.06	13.99 ± 1.09	14.35 ± 1.04	13.76 ± 1.01	14.03 ± 1.07	14.31 ± 0.99
Menton-sellion length	**11.75 ± 0.89**	**11.89 ± 1.28**	11.73 ± 1.07	11.85 ± 1.08	11.95 ± 0.99	11.66 ± 1.14	11.75 ± 0.99	11.97 ± 1.27	11.92 ± 1.07	11.67 ± 1.06	11.75 ± 1.08	12.09 ± 1.00
Bigonial breadth	11.44 ± 1.28	11.27 ±1.19	11.29 ± 1.33	11.41 ± 1.19	11.38 ± 1.22	11.36 ± 1.26	11.39 ± 1.25	11.30 ± 1.23	11.66 ± 1.30	11.02 ± 1.06	11.34 ± 1.25	11.49 ± 1.18
Head breadth	15.60 ± 0.74	15.63 ± 0.80	15.54 ± 0.80	15.65 ± 0.74	15.72 ± 0.80	15.50 ± 0.70	15.65 ± 0.74	15.51 ± 0.81	15.77 ± 0.77	15.42 ± 0.71	15.59 ± 0.75	15.73 ± 0.81
Interpupillary breadth	**6.45 ± 0.44**	**6.54 ± 0.74**	6.50 ± 0.64	6.49 ± 0.56	6.60 ± 0.59	6.38 ± 0.57	6.52 ± 0.62	6.40 ± 0.46	6.55 ± 0.56	6.42 ± 0.61	6.48 ± 0.57	6.56 ± 0.66
Frontal breadth	9.66 ± 0.99	9.90 ± 1.38	9.97 ± 1.12	9.64 ± 1.20	**9.84 ± 1.31**	**9.67 ± 1.02**	9.81 ± 1.25	9.62 ± 0.94	9.80 ± 1.14	9.71 ± 1.22	9.71 ± 1.15	10.02 ± 1.28
Nasal root width	1.77 ± 0.20	1.79 ± 0.25	1.78 ± 0.23	1.78 ± 0.22	1.80 ± 0.24	1.74 ± 0.19	1.79 ± 0.23	1.73 ± 0.20	1.78 ± 0.24	1.76 ± 0.20	1.76 ± 0.22	1.84 ± 0.24
Nose breadth	**4.16 ± 0.37**	**4.24 ± 0.64**	4.22 ± 0.54	4.19 ± 0.48	4.25 ± 0.50	4.15 ± 0.50	4.23 ± 0.54	4.10 ± 0.37	4.26 ± 0.50	4.13 ± 0.49	4.18 ± 0.49	4.29 ± 0.56
Nose protrusion	1.73 ± 0.20	1.69 ± 0.23	1.73 ± 0.25	1.70 ± 0.20	1.75 ± 0.21	1.67 ± 0.22	1.71 ± 0.22	1.69 ± 0.20	1.74 ± 0.23	1.67 ± 0.19	1.71 ± 0.22	1.71 ± 0.19
Subnasale—sellion	4.88 ± 0.48	4.83 ± 0.47	**4.87 ± 0.55**	**4.86 ± 0.43**	4.95 ± 0.42	4.77 ± 0.51	4.86 ± 0.50	4.88 ± 0.38	4.87 ± 0.52	4.85 ± 0.41	4.84 ± 0.49	4.95 ± 0.39
**Male (N = 58)**	**Cup A**		**Cup B**		**Trifold A**		**Trifold B**		**Duckbill A**		**Duckbill B**	
**Dimension (cm) mean±SD**	**Pass (n = 33)**	**Fail (n = 25)**	**Pass (n = 22)**	**Fail (n = 36)**	**Pass (n = 36)**	**Fail (n = 22)**	**Pass (n = 45)**	**Fail (n = 13)**	**Pass (n = 32)**	**Fail (n = 26)**	**Pass (n = 47)**	**Fail (n = 11)**
Bizygomatic breadth	14.57 ± 0.84	14.68 ± 1.16	14.91 ± 0.90	14.44 ± 1.00	14.63 ± 0.92	14.60 ± 1.10	14.62 ± 0.96	14.61 ± 1.11	14.75 ± 1.01	14.45 ± 0.94	14.57 ± 1.02	14.83 ± 0.84
Menton-sellion length	12.40 ± 0.64	12..3 ± 1.32	12.58 ± 0.77	12.52 ± 1.13	12.49 ± 0.91	12.63 ± 1.14	12.41 ± 0.88	13.03 ± 1.27	12.62 ± 0.98	12.44 ± 1.04	12.52 ± 1.01	12.64 ± 0.99
Bigonial breadth	12.14 ± 0.88	12.07 ± 1.07	12.42 ± 0.11	11.92 ± 0.86	12.11 ± 0.86	12.11 ± 1.11	12.17 ± 0.94	11.90 ± 1.03	12.34 ± 0.98	11.82 ± 0.87	12.08 ± 0.99	12.25 ± 0.85
Head breadth	15.98 ± 0.63	16.01 ± 0.80	16.10 ± 0.73	15.92 ± 0.69	16.09 ± 0.69	15.83 ± 0.72	15.98 ± 0.69	16.03 ± 0.77	16.05 ± 0.75	15.91 ± 0.65	15.92 ± 0.70	16.30 ± 0.67
Interpupillary breadth	**6.74 ± 0.35**	**6.95 ± 0.87**	6.92 ± 0.72	6.78 ± 0.59	6.94 ± 0.56	6.66 ± 0.72	6.88 ± 0.69	6.67 ± 0.37	6.86 ± 0.61	6.79 ± 0.68	6.80 ± 0.66	6.98 ± 0.56
Frontal breadth	9.87 ± 1.04	10.53 ± 1.61	10.39 ± 1.29	10.02 ± 1.38	10.23 ± 1.43	10.05 ± 1.23	10.16 ± 1.46	10.15 ± 0.90	10.00 ± 1.30	10.36 ± 1.41	10.06 ± 1.34	10.61 ± 1.07
Nasal root width	**1.84 ± 0.20**	**1.85 ± 0.29**	1.87 ± 0.23	1.83 ± 0.25	1.89 ± 0.25	1.78 ± 0.22	1.88 ± 0.24	1.74 ± 0.20	1.87 ± 0.24	1.82 ± 0.24	1.82 ± 0.23	1.97 ± 0.23
Nose breadth	**4.36 ± 0.38**	**4.64 ± 0.71**	4.59 ± 0.58	4.42 ± 0.55	4.51 ± 0.50	4.44 ± 0.65	4.52 ± 0.60	4.33 ± 0.35	4.57 ± 0.51	4.38 ± 0.61	4.45 ± 0.56	4.63 ± 0.56
Nose protrusion	1.77 ± 0.19	1.79 ±0.23	1.80 ± 0.24	1.77 ± 0.18	1.78 ± 0.20	1.77 ± 0.22	1.78 ± 0.22	1.78 ± 0.17	1.82 ± 0.20	1.73 ± 0.20	1.79 ± 0.21	1.72 ± 0.16
Subnasale-sellion	5.13 ± 0.33	4.99 ± 0.48	**5.14 ± 0.51**	**5.02 ± 0.33**	5.09 ± 0.39	5.03 ± 0.44	5.08 ± 0.43	5.00 ± 0.29	5.09 ± 0.45	5.04 ± 0.35	5.08 ± 0.42	5.01 ± 0.33
**Female (N = 77)**	**Cup A**		**Cup B**		**Trifold A**		**Trifold B**		**Duckbill A**		**Duckbill B**	
**Dimension (cm) mean±SD**	**Pass (n = 45)**	**Fail (n = 32)**	**Pass (n = 28)**	**Fail (n = 49)**	**Pass (n = 33)**	**Fail (n = 44)**	**Pass (n = 54)**	**Fail (n = 23)**	**Pass (n = 40)**	**Fail (n = 37)**	**Pass (n = 65)**	**Fail (n = 12)**
Bizygomatic breadth	13.6.3 ± 0.9.3	13.71 ± 0.96	**13.43 ± 0.63**	**13.79 ± 1.05**	13.61 ± 0.99	13.70 ± 0.90	13.67 ± 0.95	13.63 ± 0.92	14.02 ± 0.96	13.27 ± 0.73	13.64 ± 0.95	13.80 ± 0.90
Menton-sellion length	11.2.7 ± 0.7.3	11.20 ± 0.72	11.04 ±0.72	11.35 ± 0.71	11.36 ± 0.68	11.15 ± 0.75	11.19 ± 0.71	11.35 ± 0.77	11.35 ± 0.76	11.12 ± 0.67	11.19 ± 0.72	11.54 ± 0.70
Bigonial breadth	10.9.2 ± 1.2.9	10.60 ± 0.81	**10.36 ± 0.62**	**11.03 ± 1.26**	10.53 ± 1.02	10.97 ± 1.16	10.72 ± 1.08	10.95 ± 1.22	**11.09 ± 1.28**	**10.46 ± 0.80**	10.80 ± 1.15	10.72 ± 0.95
Head breadth	15.3.2 ± 0.6.9	15.32 ± 0.66	15.08 ± 0.51	15.45 ± 0.72	15.31 ± 0.73	15.33 ± 0.63	15.37 ± 0.67	15.20 ± 0.68	15.55 ± 0.72	15.06 ± 0.51	15.35 ± 0.70	15.16 ± 0.48
Interpupillary breadth	6.2.4 ±0.3.7	6.21 ± 0.38	**6.16 ± 0.25**	**6.26 ± 0.42**	6.22 ± 0.33	6.23 ± 0.40	6.22 ± 0.34	6.25 ± 0.45	6.30 ± 0.36	6.15 ± 0.38	6.24 ± 0.36	6.14 ± 0.46
Frontal breadth	9.5.0 ± 0.9.3	9.38 ± 0.90	9.63 ± 0.84	9.35 ± 0.95	9.42 ± 1.02	9.48 ± 0.85	9.51 ± 0.95	9.31 ± 0.83	9.63 ± 0.99	9.25 ± 0.79	9.45 ± 0.92	9.43 ± 0.91
Nasal root width	1.7.2 ± 0.1.9	1.73 ± 0.20	1.70 ± 0.20	1.74 ± 0.19	1.72 ± 0.22	1.73 ± 0.18	1.72 ± 0.19	1.73 ± 0.21	1.72 ± 0.22	1.73 ± 0.16	1.73 ± 0.20	1.71 ± 0.16
Nose breadth	4.0.2 ± 0.2.9	3.92 ± 0.33	3.91 ± 0.21	4.01 ± 0.34	3.95 ± 0.31	4.00 ± 0.30	3.99 ± 0.30	3.96 ± 0.32	4.01 ± 0.33	3.95 ± 0.28	3.98 ± 0.31	3.96 ± 0.32
Nose protrusion	1.7.0 ± 0.2.1	1.60 ± 0.20	1.68 ± 0.24	1.64 ± 0.19	1.71 ± 0.21	1.62 ± 0.20	1.66 ± 0.22	1.64 ± 0.20	**1.67 ± 0.24**	**1.64 ± 0.17**	1.65 ± 0.21	1.71 ± 0.22
Subnasale-sellion	4.7.0 ± 0.4.9	4.71 ± 0.44	4.65 ± 0.49	4.73 ± 0.45	4.80 ± 0.41	4.63 ± 0.50	4.66 ± 0.48	4.81 ± 0.42	4.69 ± 0.52	4.72 ± 0.41	4.67 ± 0.46	4.89 ± 0.46

**Bold** indicate significant T-test; p < 0.05 (S1 Table)

For male subjects, lower means of interpupillary breadth (t = -1.254; p = 0.003), nasal root width (t = -0.222; p = 0.035) and nose breadth (t = -1.957; p = 0.025) were seen in those who passed the test for Cup A. Meanwhile, higher means of subnasale-sellion (t = 1.210; p = 0.024) were reported for Cup B. Female participants who passed the test with Cup B, had significantly lower means of bizygomatic (t = -1.618; p = 0.002), bigonial breadth (t = -2.148; p = 0.005) and interpupillary breadth (t = -0.844; p = 0.010) compared to those who failed. In contrast, higher means bigonial breadth (t = 2.622; p = 0.032) and nose protrusion (t = 0.799; p = 0.012) were seen in those who passed the test for Duckbill A.

Correlations between facial dimensions and respirator fit varied among FFR models and gender are shown in Table 5. Bizygomatic breadth, bigonial breadth, head breadth and nose protrusion showed weak positive correlation with respirator fit for participants wearing Duckbill A. Whereas head breadth, interpupillary breadth, nose protrusion and subnasale-sellion had weak positive correlation with Trifold A. With positive correlations, an increase in the facial dimensions showed a better respirator fit for the specific models. For male participants, bigonial breadth were positively correlated with respirator fit of Cup B and Duckbill A model. Meanwhile for female participants, bizygomatic breadth, bigonial breadth, and head breadth had weak positive correlation with respirator fit of Duckbill A. An increase in nose protrusion and a decrease in bigonial breadth seemed to improve respirator fit for Cup A and Cup B respectively. Of note, none of the twelve facial dimensions were correlated with respirator fit for Trifold B and Duckbill B for all participants and genders.

**Table 5:** Correlation of facial dimension with overall fit factor based on N95 models.

**Total Participants, N:135**						
**Facial Dimensions**	**Cup A**	**Cup B**	**Trifold A**	**Trifold B**	**Duckbill A**	**Duckbill B**
Bizygomatic breadth	-0.061	0.062	0.069	0.054	0.279**	-0.052
Menton-sellion length	-0.055	0.029	0.156	-0.082	0.124	-0.075
Bigonial breadth	0.039	-0.020	0.015	0.044	0.271**	-0.041
Head breadth	-0.047	-0.018	0.169*	0.151	0.288**	-0.046
Interpupillary breadth	-0.054	0.017	0.174*	0.112	0.125	-0.066
Frontal breadth	-0.082	0.007	0.060	0.070	0.040	-0.097
Nasal root width	0.021	0.001	0.126	0.124	0.071	-0.117
Nose breadth	-0.057	0.049	0.139	0.103	0.152	-0.086
Nose protrusion	0.086	0.156	0.170*	0.041	0.188*	-0.034
Subnasale-sellion	0.067	0.115	0.185*	-0.050	0.010	-0.039
**Male, N: 58**						
**Facial Dimensions**	**Cup A**	**Cup B**	**Trifold A**	**Trifold B**	**Duckbill A**	**Duckbill B**
Bizygomatic breadth	-0.066	0.249	0.029	0.026	0.083	0.033
Menton-sellion length	-0.199	0.074	-0.026	-0.239	0.083	0.033
Bigonial breadth	-0.010	0.263*	-0.023	0.117	0.341**	-0.105
Head breadth	-0.096	0.111	0.166	0.011	0.202	-0.187
Interpupillary breadth	-0.117	0.080	0.214	0.140	0.078	-0.062
Frontal breadth	-0.193	0.035	0.034	-0.005	-0.113	-0.143
Nasal root width	0.111	0.042	0.237	0.246	0.132	-0.204
Nose breadth	-0.213	0.150	0.103	0.128	0.181	-0.096
Nose protrusion	-0.120	0.124	0.084	-0.035	0.225	0.136
Subnasale-sellion	0.090	0.209	0.099	0.055	0.015	0.129
**Female, N:77**						
**Facial Dimensions**	**Cup A**	**Cup B**	**Trifold A**	**Trifold B**	**Duckbill A**	**Duckbill B**
Bizygomatic breadth	-0.090	-0.132	-0.050	0.020	0.389**	0.028
Menton-sellion length	0.035	-0.096	0.153	-0.091	0.181	-0.090
Bigonial breadth	0.072	-0.277*	-0.156	-0.073	0.264*	0.087
Head breadth	-0.036	-0.178	0.046	0.222	0.385**	0.149
Interpupillary breadth	-0.026	-0.142	-0.062	0.024	0.198	0.035
Frontal breadth	0.019	0.096	-0.028	0.111	0.193	0.010
Nasal root width	-0.075	-0.071	-0.073	0.022	0.001	0.012
Nose breadth	0.106	-0.156	-0.010	0.015	0.137	0.023
Nose protrusion	0.239*	0.163	0.158	0.059	0.156	-0.124
Subnasale-sellion	0.046	0.028	0.148	-0.175	-0.015	-0.100

**Significant at the 0.01 level (2-tailed) using Pearson correlation

*Significant at the 0.05 level (2-tailed) using Pearson correlation

Generalised linear regression with gamma log link was performed to assess the direction and magnitude of effect between various facial dimensions and overall fit factor for different N95 models (S2 Table). From the analysis, only nose protrusion showed a positive effect on overall fit factor in total participants (exp(β) = 2.488, p = 0.041) for Duckbill A and females (exp(β) = 2.929, p = 0.040) for Cup A. Meanwhile in males, overall fit factor increases as nasal root (exp(β) = 2.946, p = 0.032) and subnasale-sellion (exp(β) = 1.998, p = 0.023) increases for Cup A. This is in contrast with menton-sellion (exp(β) = 0.775, p = 0.013) and nose breadth (exp(β) = 0.553, p = 0.025) with negative effect on fit factor. Meanwhile, a positive effect can be seen with menton-sellion (exp(β) = 0.820, p = 0.025) for Trifold B and a negative effect of bigonial breadth (exp(β) = 1.474, p = 0.024) for Duckbill A. Bizygomatic breadth, head breadth, interpupillary breadth and frontal breadth had no significant effect with fit factor for any of the FFR.

## Discussion

In recent years, several infectious disease outbreaks such as the SARS, influenza, H1N1, MERS, and, most recently, the COVID-19 pandemic, have demonstrated transmission from infected patients to healthcare workers, resulting in severe debilitating illness and death [40–42]. These alarming events warrant a high level of protection against transmission of airborne diseases through the recommended use of PPE, particularly respirators. Generally, a single type or size of FFR is not sufficient to protect individuals with different anthropometric dimensions [43, 44]. Fit testing allows individuals with different facial morphologies to be tested with the available respirators on the market.

Overall, the passing rates for N95 in this study were between 36.3% and 83.7%. This was comparable with a study among healthcare workers in Australia with overall fit test passing rates between 32.4% and 96.4% [45]. Another study found lower fit tests passing rates (0–43.2%), with 17 out of 20 FFR had less than 20% passing rate [10]. A study among 50 healthcare providers using nine different FFR found that fit test passing rates varied significantly depending on the design [43]. They found that the flat-fold N95 had a higher rate (57.5%) of successful fit tests than duckbill (18.3%) and hard-shell (cup) designs (3.3%). However, our study showed that the duckbill (Duckbill B) was superior than the three-panel-flat-fold and cup designs. While the passing rate for cup designs in our study is the lowest (36.3–57.0%), it was comparable to a study by Zhang et al., in 2020 with 52.9%, Fakherpour et al., 2021 (0–43.2%) and Coffey et al., 2004 (0–43.8%) [10, 32, 46]. The results of our study are also consistent with some other studies [21, 45, 47].

It is also important to note that FFR with similar designs can have marked differences in pass rates, as shown in our study for cup, trifold, and duckbill designs (Table 2). Compared to other studies, our findings demonstrated a GM FF_Ο_ of 37.29–79.64 for the cup design, which is higher than Zhuang et al., 2005 (7.00–52.00), and lower than Zhang et al., 2020 (112.50) [32, 48]. The GM FF_Ο_ for trifold design in our study was 120.16 for Trifold B and 79.60 for Trifold A when compared to the fold design in their study with 92.2 GM FF_Ο_. The fold design FFR in our study also demonstrated higher passing rates (50.4–74.1%) compared to Zhang et al. with 40.0% [32]. The difference in passing rate and fit factor is expected although they have similar designs, they were made in different countries which may tailored more to specific populations with different range of facial dimensions. For example, the mean bizygomatic breadth for participants who passed the test for the cup design in Zhang et al., 2020 were 11.20±0.63 cm, compared to our participant who passed the test for Cup A (14.03±1.00 cm) [32].

Most N95 in this study showed a better fit for male participants compared to female participants, with higher passing rates for Cup B, Trifold A, Trifold B and Duckbill A. This is probably because, most male participants have large facial sizes (50.0%) and have larger mean for all facial dimensions which possibly explained the better fit for most of the FFR studied. The finding is also consistent with other previous publications which demonstrated a generally better fit for FFR in male participants than female participants [20, 28, 49]. Meanwhile, Cup A and Duckbill B in this study, showed higher passing rates in participants who have smaller mean of facial dimensions which are mostly females (Table 4). A study among Australian healthcare workers reported that female participants had a higher pass rate (59.9%) compared to male participants (48.1%) [50]. Another study also showed higher passing rates in female participants (13.5%) compared to males (2.7%), but the markedly low passing rates is unlikely able to represent other population [51]. It is also worth noted that FFR with the highest passing rates, (Duckbill B and Trifold B) showed no significant difference (p>0.05) of facial dimensions between pass or fail (Table 4). This is likely due to the better design with soft material and a nose foam that conforms to the contour of the face and the shape of the nasal bridge providing a better face seal to prevent leakage.

The result of this study also demonstrated that some aspects of facial dimensions are important in determining facial fit to respirators. T-test results on the facial dimensions showed significant differences for ‘pass’ and ‘fail’ results when wearing Cup A, Cup B and Trifold A. Menton-sellion length (t = -0.736, p = 0.027), interpupillary breadth (t = -0.844, p = 0.037), and nose breadth (t = -0.893, p <0.001) seemed to be significant in determining the pass or fail result for Cup A. Meanwhile frontal breadth (t = 0.847, p = 0.024) and subnasale-sellion (t = 0.202, p = 0.007) were significant for Trifold A and Cup B respectively. This is in contrast with the study by Zhang et al. [32], where no significance was found for any facial dimensions when wearing cup designed FFR. However, in the fold designed FFR, morphological facial length, nose height and nose length (t = 4.525, -2.738, and -2.725, p<0.05) were found to be significant between passed and failed participants [32]. Thus, emphasising that fit test performance for each FFR model were related to different aspect of facial dimensions.

In this study, bigonial breadth, head breadth and bizygomatic breadth (p<0.01), and interpupillary breadth, subanasale sellion and nose protrusion (p<0.05) have significant correlations with respirator fit (Table 5). In contrast, Zhang et al.’s study showed only morphological facial length demonstrate significant correlation with fit factor [32]. Oestenstad et al., 2007 reported a positive correlation with bizygomatic breadth (r = 0.275, p<0.05) and bigonial breadth (r = 0.385, p<0.05) in females [17] which is similar to our study with (r = 0.389, p<0.01) and (r = 0.385, p<0.01) respectively. They also highlighted the difference between genders where out of twelve facial dimensions, only biectoorbital breadth and bizygomatic breadth had significant correlations for the same N95 in both genders [17]. Generalised linear regression for our study showed that only nose protrusion had a significant effect with overall fit factor for all participants for one N95, Duckbill A. This is in contrast with studies by Zhuang et al. and Oestenstad et al., whereby all twelve measured facial dimensions have significant regression coefficient with fit factors [17, 48]. Comparison of facial panels showed that the mean facial width for Malaysian male (141.5mm) was different compared to the US NIOSH respirator study (143.5mm), but the same in female (135.1mm). Meanwhile, the Malaysian facial panel was different in both male and female compared to the Chinese population (male: 147.5mm, female: 139.9mm) [30, 52, 53]. These results highlight the differences of facial anthropometry among different populations and ethnicity.

FFR of different shapes and sizes with good fit should be made available in healthcare facilities particularly the duckbill and trifold FFR which have been shown to give more protection compared to the cup design [43]. The duckbill and trifold FFR’s materials and designs used in this study are easily moulded which ensure air-tightness that contours better with the participants faces, compared to rigid and unbent cup N95, which are more prone to leakage. Overall, the key strength of this study is that the FFR selected were the most used in our healthcare settings. The use of quantitative fit testing can prevent perception bias as it objectively measures the amount of leakage (quantity) compared to qualitative testing that relies subjectively on the users taste and smell to detect leakage. In addition, the facial size (small, medium and large) categorised in this study is based on a large nationwide survey of head and facial anthropometry of diverse ethnic groups in Malaysia, which included over 3000 individuals [30]. However, our study did not compare passing rates between the major ethnicities in Malaysia (i.e., Malays, Chinese and Indians). The current study also has a limitation in selecting N95 due to limited availability between brands, models, and sizes. The researchers were unable to identify all FFR with different sizes, i.e., only one FFR model was available in two sizes, while the rest were in universal size, making comparison difficult.

## Conclusion

In conclusion, the low passing rate for certain N95 designs from the fit test in this study, gives insight on the importance and the requirement to select proper FFR in terms of size, type, and models. The duckbill and trifold design showed the best passing rates across all demographics and facial sizes compared to cup design. In general, certain FFR model (Cup B, Trifold, A, Trifold B, Duckbill A) fit better for participants with large facial size, while others (Cup A and Duckbill B) specifically fit better for those with small facial size. This results also reflects similarly for gender differences as most males have large facial size while most females have small facial size. However, two FFR (Trifold B and Duckbill B) performed better than other FFR across all demographics and facial sizes, with passing rate of more than 70%. We found the positive effect of nose protrusion, nasal root and subnasale-sellion and the negative effect of menton-sellion, bigonial breadth and nose breadth on fit factors. Therefore, it is important to note that, not all FFR fit the same and that the choice for FFR should consider these differences. Annual fit testing for employees also needs to be reinforced as per Ministry of Health’s policy for infection prevention and control in ensuring safe respirator option in high-risk settings.

## Supporting information

S1 Table Independent sample T-test for facial dimensions of participants according to ‘pass’ and ‘fail’ test result for each N95 respirators. (DOCX)

S2 Table Generalised linear regression of facial dimensions to overall fit factor based on N95. (DOCX)
