# Supplementary material for: The effect of N95 designs on respirator fit and its associations with gender and facial dimensions
Source: PLoS One. 2023 Nov 29;18(11):e0288105. doi: 10.1371/journal.pone.0288105 (PMC10686483; doi:10.1371/journal.pone.0288105)
Supplement: S1 Table — (DOCX) [file pone.0288105.s001.docx]

**S1 Table. Independent sample T-test for facial dimensions of participants according to ‘pass’ and ‘fail’ test result for each N95 respirators.**

| **TOTAL** | **Cup A**  **t test (p value)** | **Cup B**  **t test (p value)** | **Trifold A**  **t test (p value)** | **Trifold B**  **t test (p value)** | **Duckbill A**  **t test (p value)** | **Duckbill B**  **t test (p value)** |
| --- | --- | --- | --- | --- | --- | --- |
| Bizygomatic |  |  |  |  |  |  |
| Menton-Sellion | -0.736 (0.027) |  |  |  |  |  |
| Bigonial breadth |  |  |  |  |  |  |
| Head breadth |  |  |  |  |  |  |
| Interpupillary breadth | -0.844 (0.037) |  |  |  |  |  |
| Frontal breadth |  |  | 0.847 (0.024) |  |  |  |
| Nasal root width |  |  |  |  |  |  |
| Nose breadth | -0.893 (<0.001) |  |  |  |  |  |
| Nose protrusion |  |  |  |  |  |  |
| Subnasale - Sellion |  | 0.202 (0.007) |  |  |  |  |
| **FEMALE** | **Cup A**  **t test (p value)** | **Cup B**  **t test (p value)** | **Trifold A**  **t test (p value)** | **Trifold B**  **t test (p value)** | **Duckbill A**  **t test (p value)** | **Duckbill B**  **t test (p value)** |
| Bizygomatic |  | -1.618 (0.002) |  |  |  |  |
| Menton-Sellion |  |  |  |  |  |  |
| Bigonial breadth |  | -2.418 (0.005) |  |  | 2.622 (0.032) |  |
| Head breadth |  |  |  |  |  |  |
| Interpupillary breadth |  | -0.844 (0.010) |  |  |  |  |
| Frontal breadth |  |  |  |  |  |  |
| Nasal root width |  |  |  |  |  |  |
| Nose breadth |  |  |  |  |  |  |
| Nose protrusion |  |  |  |  | 0.799 (0.012) |  |
| Subnasale - Sellion |  |  |  |  |  |  |
| **MALE** | **Cup A**  **t test (p value)** | **Cup B**  **t test (p value)** | **Trifold A**  **t test (p value)** | **Trifold B**  **t test (p value)** | **Duckbill A**  **t test (p value)** | **Duckbill B**  **t test (p value)** |
| Bizygomatic |  |  |  |  |  |  |
| Menton-Sellion |  |  |  |  |  |  |
| Bigonial breadth |  |  |  |  |  |  |
| Head breadth |  |  |  |  |  |  |
| Interpupillary breadth | -1.254 (0.003) |  |  |  |  |  |
| Frontal breadth |  |  |  |  |  |  |
| Nasal root width | -0.222 (0.035) |  |  |  |  |  |
| Nose breadth | -1.957 (0.025) |  |  |  |  |  |
| Nose protrusion |  |  |  |  |  |  |
| Subnasale - Sellion |  | 1.210 (0.024) |  |  |  |  |

Only significant t-test are display in this table

Significant t-test: p < 0.05
