# Supplementary material for: The effect of N95 designs on respirator fit and its associations with gender and facial dimensions
Source: PLoS One. 2023 Nov 29;18(11):e0288105. doi: 10.1371/journal.pone.0288105 (PMC10686483; doi:10.1371/journal.pone.0288105)
Supplement: S2 Table — (DOCX) [file pone.0288105.s002.docx]

**S2 Table. Generalised linear regression of facial dimensions to overall fit factor based on N95.**

| **TOTAL** | | | | | | |
| --- | --- | --- | --- | --- | --- | --- |
| **Facial dimensions** | **Cup A** | **Cup B** | **Trifold A** | **Trifold B** | **Duckbill A** | **Duckbill B** |
| Bizygomatic | 0.943 | 1.265 | 0.906 | 0.975 | 1.183 | 0.998 |
| Menton-sellion | 0.835 | 0.916 | 0.951 | 0.870 | 1.019 | 0.994 |
| Bigonial breadth | 1.114 | 0.895 | 0.914 | 0.990 | 1.061 | 1.014 |
| Head breadth | 1.036 | 0.901 | 1.300 | 1.137 | 1.067 | 0.975 |
| Interpupillary breadth | 0.934 | 0.851 | 1.189 | 1.018 | 0.969 | 1.088 |
| Frontal breadth | 0.974 | 1.067 | 0.983 | 0.992 | 0.933 | 0.966 |
| Nasal root | 1.567 | 1.071 | 1.223 | 1.282 | 0.843 | 0.799 |
| Nose breadth | 0.857 | 1.109 | 1.053 | 1.078 | 0.964 | 0.963 |
| Nose protrusion | 1.384 | 1.669 | 1.158 | 1.136 | **2.488** | 1.004 |
| Subnasale-sellion | 1.345 | 1.179 | 1.330 | 1.113 | 0.727 | 0.968 |
| **FEMALE** | | | | | | |
| **Facial dimensions** | **Cup A** | **Cup B** | **Trifold A** | **Trifold B** | **Duckbill A** | **Duckbill B** |
| Bizygomatic | 0.798 | 1.203 | 0.929 | 1.006 | 1.299 | 0.942 |
| Menton-sellion | 1.048 | 1.172 | 1.342 | 1.097 | 1.714 | 0.912 |
| Bigonial breadth | 1.119 | 0.694 | 0.818 | 0.886 | 0.947 | 1.042 |
| Head breadth | 1.006 | 0.865 | 1.202 | 1.243 | 1.188 | 1.120 |
| Interpupillary breadth | 0.971 | 0.771 | 1.108 | 0.926 | 1.288 | 0.966 |
| Frontal breadth | 1.018 | 1.215 | 1.005 | 1.059 | 1.115 | 0.999 |
| Nasal root | 0.853 | 1.819 | 0.880 | 0.999 | 0.591 | 0.958 |
| Nose breadth | 1.502 | 0.596 | 1.009 | 1.025 | 0.509 | 1.032 |
| Nose protrusion | **2.929** | 2.533 | 1.281 | 1.338 | 2.581 | 0.801 |
| Subnasale-sellion | 0.834 | 0.749 | 0.906 | 0.709 | 0.490 | 1.094 |
| **MALE** | | | | | | |
| **Facial dimensions** | **Cup A** | **Cup B** | **Trifold A** | **Trifold B** | **Duckbill A** | **Duckbill B** |
| Bizygomatic | 1.084 | 1.260 | 0.889 | 0.966 | 1.067 | 1.125 |
| Menton-sellion | **0.775** | 0.940 | 0.873 | **0.820** | 1.034 | 0.996 |
| Bigonial breadth | 1.158 | 1.222 | 1.004 | 1.102 | **1.474** | 0.967 |
| Head breadth | 0.936 | 0.939 | 1.327 | 1.044 | 0.970 | 0.832 |
| Interpupillary breadth | 1.211 | 0.816 | 1.455 | 1.119 | 0.827 | 1.183 |
| Frontal breadth | 0.891 | 0.930 | 0.949 | 0.944 | 0.810 | 0.941 |
| Nasal root | **2.946** | 1.319 | 1.593 | 1.846 | 1.247 | 0.614 |
| Nose breadth | **0.553** | 1.442 | 0.861 | 0.983 | 1.438 | 0.948 |
| Nose protrusion | 0.585 | 0.676 | 0.773 | 0.714 | 3.199 | 1.446 |
| Subnasale-sellion | **1.998** | 2.459 | 1.594 | 1.662 | 0.986 | 0.883 |

**Bold** indicate significant Exponential Beta coefficient (p < 0.05).
